# Dynamic susceptibility of Fe_3_O_4_ nanotubes

**DOI:** 10.1186/s11671-023-03841-5

**Published:** 2023-04-07

**Authors:** Enzo Fabrizio Pusiol, Eduardo Saavedra, Alejandro Pereira, Juan Luis Palma, Noelia Bajales, Juan Escrig

**Affiliations:** 1 FAMAF, Universidad Nacional de Córdoba, 5000 Córdoba, Argentina; 2 Department of Physics, University of Santiago de Chile (USACH), 9170124 Santiago, Chile; 3 Department of Sciences, Faculty of Liberal Arts, Adolfo Ibáñez University, 7941169 Santiago, Chile; 4 School of Engineering, Central University of Chile, 8330601 Santiago, Chile; 5 Center for the Development of Nanoscience and Nanotechnology (CEDENNA), 9170124 Santiago, Chile; 6 CONICET, IFEG, Av. Medina Allende s/n, 5000 Córdoba, Argentina

## Abstract

In this work we performed a detailed numerical analysis to investigate the dynamic susceptibility of 1000 nm long Fe_3_O_4_ nanotubes by varying the diameter, the tube wall thickness and the magnitude of the external magnetic field applied along the tube axis. We found two well-defined modes, one of low frequency associated with the caps of the nanotubes, and another of high frequency associated with the central area of the nanotubes, which can be controlled by varying the geometry of the tubes or the external magnetic field to which they are subjected. These results allow us to suggest the use of these nanotubes in applications that require controlling the resonant frequency in the GHz range.

**Supplementary Information:**

The online version contains supplementary material available at 10.1186/s11671-023-03841-5.

## Introduction

Magnetic nanostructures are very important for several modern applications such as sensors, spintronics, information storage devices, microwave devices, and biomedicine [1–6]. Among these, one of the most intensely investigated are magnetic nanotubes, because they exhibit a high aspect ratio, like nanowires, but with two functionalizable surfaces [7], in addition to offering a lightweight alternative to design mechanical nanodevices with minimal loss of mechanical performance [8]. Furthermore, these tubes allow the mobility of domain walls [9] and skyrmionic structures [10, 11], making them promising candidates as information carriers.

Ferromagnetic resonance (FMR) is a powerful technique to investigate the magnetic properties of ferromagnetic materials, which is based on applying a transverse magnetic field (microwave fields) so that the system absorbs energy at a certain resonance frequency (ω). Da Silva et al. [12, 13] have investigated the dynamic properties of hollow square nanopillars. Also, the spin wave spectra associated with a confined vortex domain wall within a nanotube has been calculated [14] as well as the nonreciprocity of spin waves in magnetic nanotubes with helical equilibrium magnetization [15] and the effects of an external magnetic field on the spin waves of a finite nanotube [16]. Fel'k et al. [17] investigated the ferromagnetic resonance of a nickel microtube, Yong et al. [18] investigated the microwave electromagnetic and absorption properties of hollow nanostructures, while Saavedra et al. had already reported the dynamic susceptibility of curved nanotubes [19] and wire-tube nanostructures [20]. Despite all these efforts to understand the dynamic properties of hollow nanostructures, there is still a lack of studies focused on the dynamic susceptibility of Fe_3_O_4_ nanotubes.

In this work we have systematically studied, through micromagnetic simulations, the dynamic susceptibility of Fe_3_O_4_ nanotubes. The main goal was to study the number of resonance modes and the frequency at which they appeared as a function of the diameter (*D*), the tube wall thickness (*W*), the magnitude of the external magnetic field (*B*) applied along the tube axis (*z*-axis) and the inter-nanotube distances in a Fe_3_O_4_ nanotube array.

## Micromagnetic simulations

The dynamic behavior of Fe_3_O_4_ nanotubes was investigated using micromagnetic simulations conducted with the Object Oriented Micromagnetic Framework (OOMMF) software [21]. OOMMF solves the Landau–Lifshitz–Gilbert equation (LLG) [22]:

$$\frac{{d\vec{M}}}{dt} = - \gamma \vec{M}{ } \times { }\vec{H}_{eff} - \frac{\gamma \alpha }{{M_{s} }}\vec{M} \times { }\left( {\vec{M} \times { }\vec{H}_{eff} } \right),$$

where $\vec{M}$ represents the magnetization, $\vec{H}_{eff}$ is the effective magnetic field, $\gamma$ denotes the gyromagnetic ratio, and *α* is the damping constant. This equation describes the precession of the magnetization around $\vec{H}_{eff}$, which results in a torque on the magnetization that is proportional to the gyromagnetic ratio. OOMMF iteratively solves this equation for each cell of a selected mesh using the finite differences method [23] and enables monitoring of the temporal evolution of the system and the dynamic behavior of its magnetization.

In this work we have considered single Fe_3_O_4_ nanotubes as well as an array of seven of them. The single nanotubes are of *L* = 1000 nm in length, because Xiang et al. [24] demonstrated that the magnetic properties of a 20 nm diameter Fe nanowire did not vary for lengths greater than 200 nm. The single nanotubes have three possible external diameters (*D* = 52, 60 and 72 nm) and a variable tube wall thickness between *W* = 5 and 30 nm (see Fig. 1 in Supplementary Information). The simulated array consists of seven parallel-aligned Fe_3_O_4_ nanotubes, each L = 1000 nm in length. These nanotubes have a diameter of *D* = 52 nm and a tube wall thickness of *W* = 15 nm. One of the nanotubes is located in the center of a hexagonal cell, while the other six are located with their easy axis on each vertex of such hexagonal cell. For the array, inter-nanotube distances, *d*_cc_, ranging from 1.0*D* to 3.0*D* were simulated, using an external magnetic field of *B* = 5 kOe. The magnetic parameters used for magnetite (Fe_3_O_4_) were the saturation magnetization M_s_ = 4.8 × 10^5^ A/m and the stiffness constant A = 1.3 × 10^−11^ J/m [25]. Polycrystalline nanotubes have been performed since magnetocrystalline anisotropy was neglected. We have used a cell size of 2 × 2 × 5 nm^3^, small enough to reproduce the geometry of the nanostructures.

To simulate the magnetic configurations of minimum energy, we have used *α* = 0.5, generally used to reduce simulation time without affecting the results of the quasi-static simulations. The external magnetic field was applied parallel (*θ* = 0°) to the *z*-axis. On the other hand, to simulate the dynamic response of the magnetization (FMR spectra), we have used a smaller value of *α* = 0.015 [26]. In addition, we have used a sinc wave excitation field [19]: $h_{sinc} = h_{0} \frac{{sin\left( {2\pi f_{c} \tau } \right)}}{{2\pi f_{c} \tau }}$, applied along the *x-*direction to perturb the magnetization of the system. The amplitude of the sinc wave was $h_{0}$ = 1 mT, the cut-off frequency $f_{c}$ = 45 GHz and *τ* = t-t_0_ was the simulation time (t), with an offset t_0_. The data in the time domain were recorded for 20 ns with a step time of 5 ps, allowing a better spectral resolution of 0.05 GHz. For FMR analysis, the imaginary part of the dynamical susceptibility is derived through a fast-Fourier transform (FFT) procedure. Specifically, the dynamic susceptibility χ(ω) at a frequency ω is defined as the ratio between the Fourier component, m_x_(ω), of the *x*-component of the spatially averaged magnetization m(t) and the Fourier component, h(ω), of the applied exciting field [27], χ(ω) = m_x_(ω)/h(ω). We have further obtained the spatial FMR mode profiles from the post processing of the position dependent magnetization data.

## Results and discussion

In this section, we show and discuss the results of our micromagnetic simulations for the dynamic properties of Fe_3_O_4_ nanotubes. We focus on the dynamic susceptibility and the resonant frequency of the peaks for the different geometric parameters and magnetic fields considered.

### Variation of the diameter (D) Fe_3_O_4_ nanotubes

**Fig. 1:**
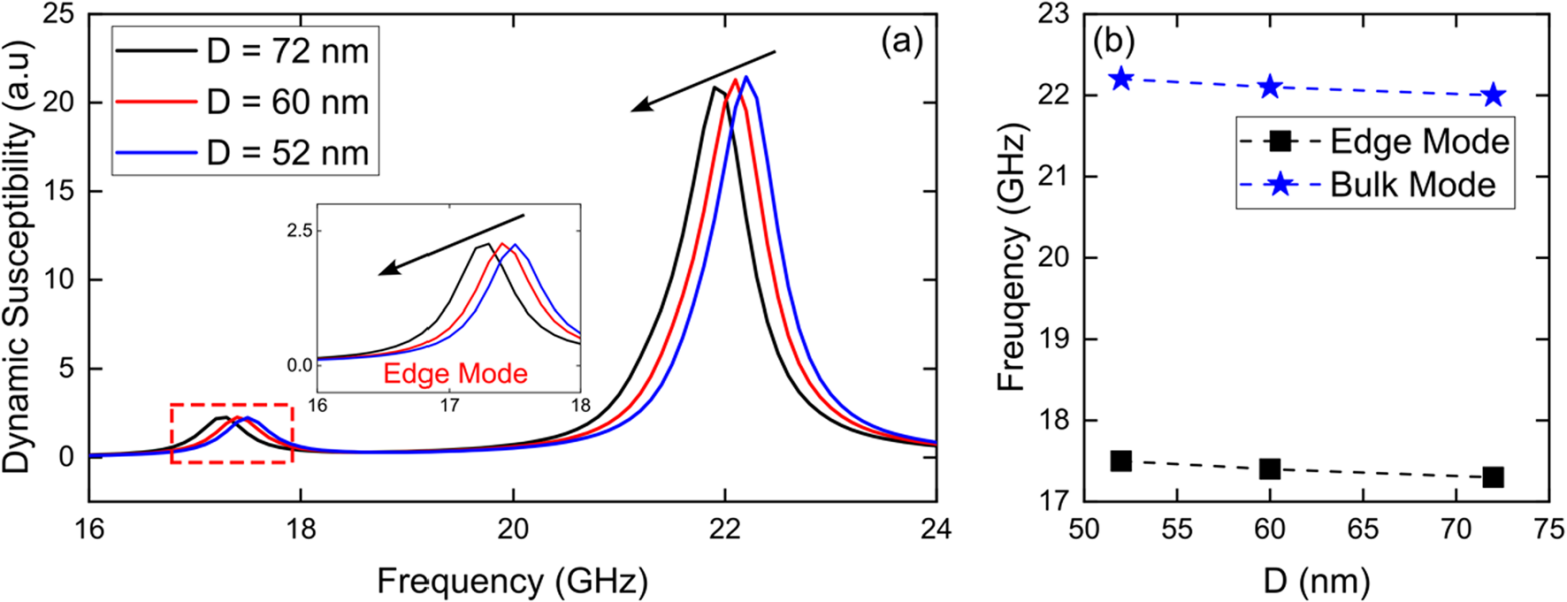
**a** Dynamic susceptibility spectra for 1000 nm long single Fe_3_O_4_ nanotubes with a tube wall thickness fixed at *W* = 15 nm and three different diameters, *D* = 52, 60 and 72 nm, by applying a sinc wave excitation in the *x*-direction in the presence of an external magnetic field of *B* = 5.0 kOe along the *z*-axis. **b** Evolution of resonance modes, edge mode (black squares) and bulk mode (blue stars), as a function of the diameters (*D*) of the nanotubes

In a first stage, we investigated the dynamic susceptibility spectra for 1000 nm long single Fe_3_O_4_ nanotubes with a tube wall thickness fixed at *W* = 15 nm and three different diameters, *D* = 52, 60 and 72 nm, by applying a sinc wave excitation in the *x*-direction in the presence of an external magnetic field of *B* = 5.0 kOe along the *z*-axis. From Fig. 1a we can see two well-defined modes, one at low frequency, edge mode, associated with the caps of the nanotube, and another at high frequency, bulk mode, of larger height, since it excites a greater number of magnetic moments associated with the central area of the nanotube (see Fig. S2 in Supplementary Information). The relative magnitudes of the peaks can be explained by the relative volumes affected in the different regions [25]. From Fig. 1b we can see how both modes slightly decrease their frequency as the diameter of the nanotubes increases. This can be explained because nanotubes are very long, so the diameters we have considered do not significantly change the aspect ratio of the tubes.

### Variation of the tube wall thickness (W) of the Fe_3_O_4_ nanotubes

**Fig. 2:**
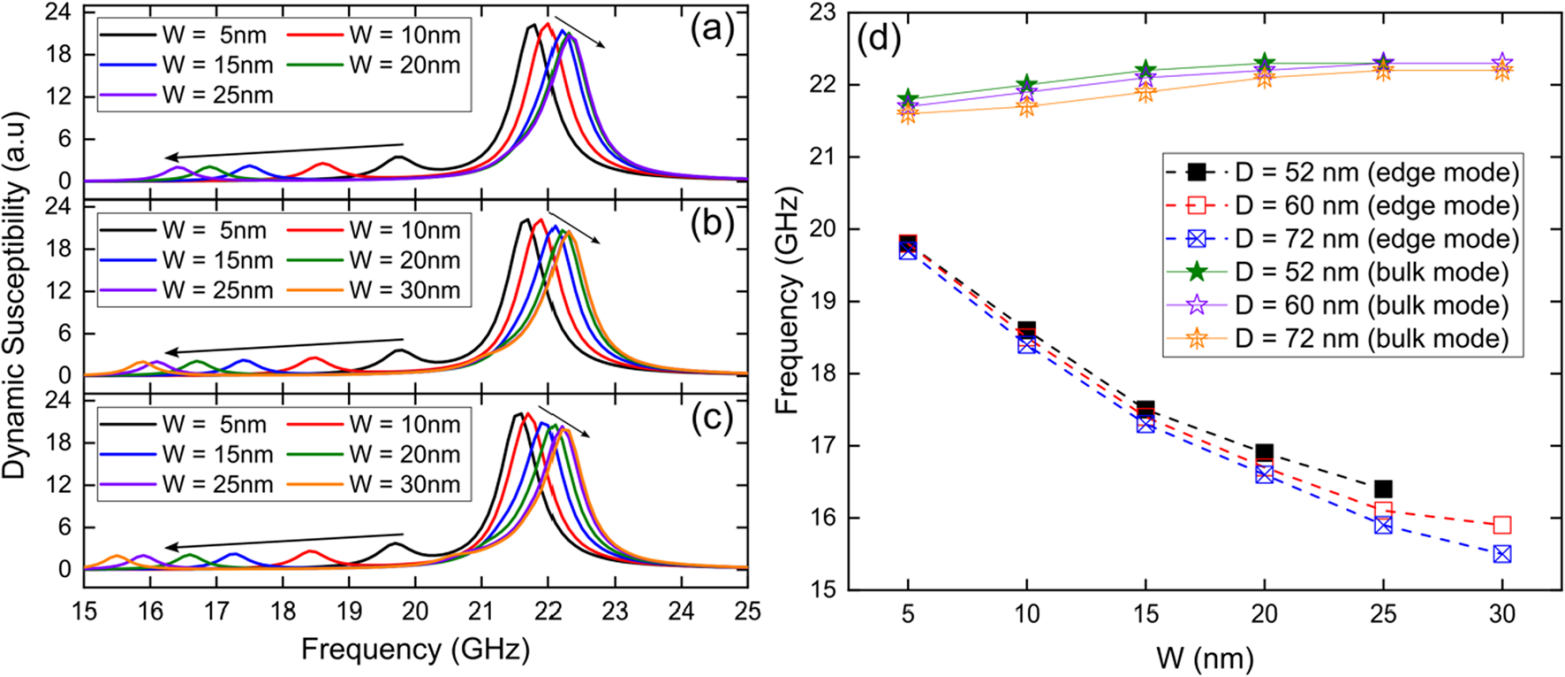
Dynamic susceptibility spectra for 1000 nm long single Fe_3_O_4_ nanotubes with three different diameters, *D* = 52 nm (**a**), 60 nm (**b**) and 72 nm (**c**), as a function of the tube wall thickness (*W*), by applying a sinc wave excitation in the *x*-direction in the presence of an external magnetic field of *B* = 5.0 kOe along the *z*-axis. (**d**) Evolution of resonance modes, edge mode (squares) and bulk mode (stars), as a function of the tube wall thickness (*W*) of the nanotubes

Next, we investigated the dynamic susceptibility spectra for 1000 nm long single Fe_3_O_4_ nanotubes with three different diameters, *D* = 52, 60 and 72 nm, as a function of the tube wall thickness (*W*), by applying a sinc wave excitation in the *x*-direction in the presence of an external magnetic field of *B* = 5.0 kOe along the *z*-axis. From Fig. 2 we can see that regardless of the diameter (*D*) and wall thickness (*W*) of the tube, nanotubes exhibit the two resonance modes already seen in Fig. 1 and described above. From Fig. 2d we can see that the edge mode significantly decreases its resonance frequency with increasing tube wall thickness (*W*), from about 20 GHz for *W* = 5 nm to 16 GHz for *W* = 30 nm.

On the other hand, the bulk mode increases slightly with increasing tube wall thickness (*W*) but remaining practically at 22 GHz for the entire range of thicknesses investigated. In this way we see how the tube wall thickness affects more intensely the magnetic moments located at the edges of the tube than those in the central zone, which is mainly due to the surfaces involved. Figure 2d again confirms that an increase in the diameter of the nanotubes produces a slight decrease in the frequencies at which the resonance modes are excited, as shown in Fig. 1b.

### Variation of the external magnetic field (B) applied to the Fe_3_O_4_ nanotubes

**Fig. 3:**
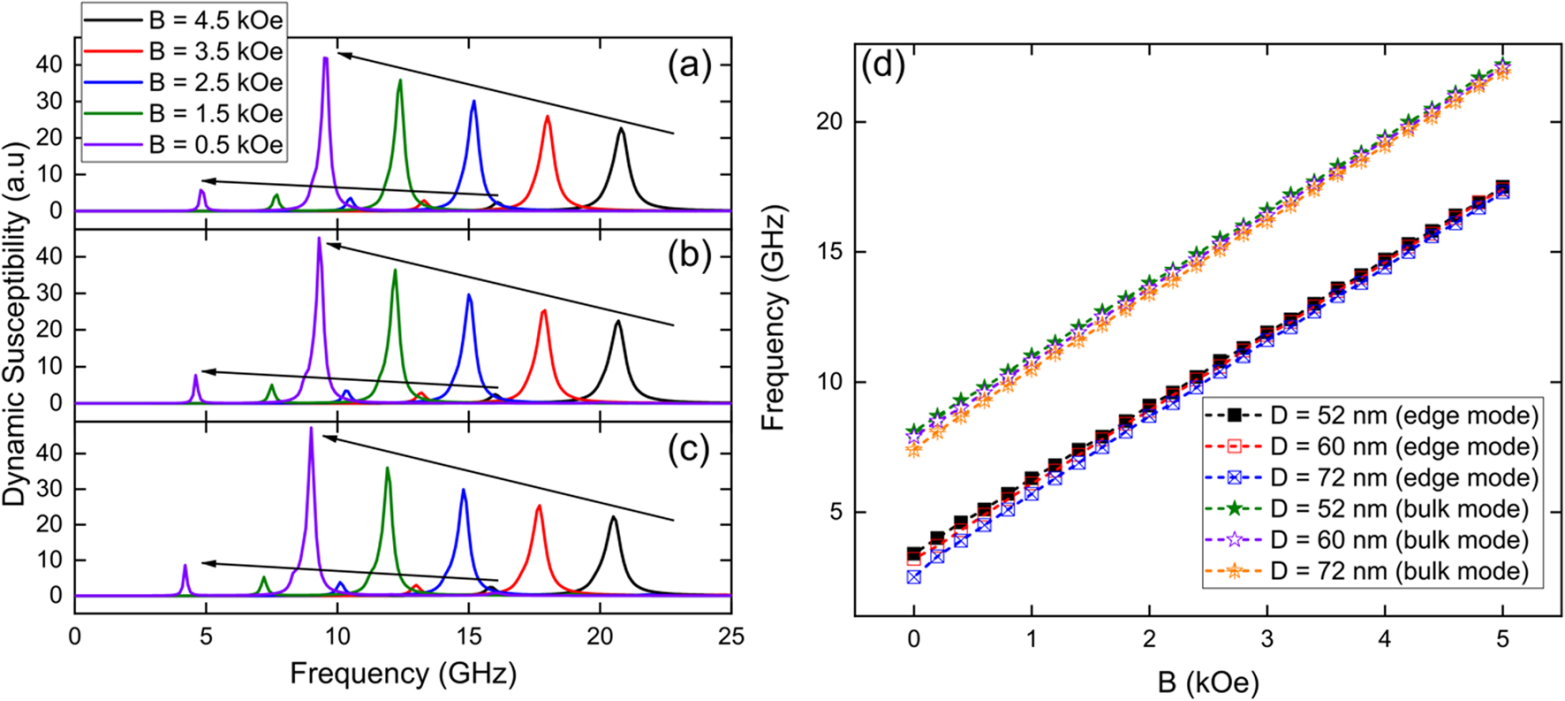
Dynamic susceptibility spectra for 1000 nm long single Fe_3_O_4_ nanotubes with a wall thickness of *W* = 15 nm, three different diameters, *D* = 52 nm (**a**), 60 nm (**b**) and 72 nm (**c**), as a function of the external magnetic field (*B*) applied along the *z*-axis, by applying a sinc wave excitation in the *x*-direction. (**d**) Evolution of resonance modes, edge mode (squares) and bulk mode (stars), as a function of the external magnetic field (*B*) applied along the *z*-axis

In addition, we investigated the dynamic susceptibility spectra for 1000 nm long single Fe_3_O_4_ nanotubes as a function of the external magnetic field (*B*) applied along the *z*-axis. In this case, we considered nanotubes with a wall thickness of *W* = 15 nm, three different diameters, *D* = 52, 60 and 72 nm, by applying a sinc wave excitation in the *x*-direction. From Fig. 3 we can see that regardless of the applied external magnetic field applied, the nanotubes still exhibit only two reversal modes for the geometric parameters investigated here. From Fig. 3d we can see that the edge mode increases its resonance frequency with increasing external magnetic field that is applied along the *z*-axis, from about 3 GHz for *B* = 0 up to 17 GHz for *B* = 5 kOe. Similarly, the bulk mode increases its resonant frequency from approximately 8 GHz for *B* = 0 to 22 GHz for *B* = 5 kOe.

From Figs. 3a, b and c we can see that in addition to increasing the frequency at which the modes appear by increasing the external magnetic field, we can also see that the height of such peaks decreases, which implies that fewer magnetic moments are being excited with the increase of the external magnetic field.

### Variation of the inter-nanotube distances in a Fe_3_O_4_ nanotube array

**Fig. 4:**
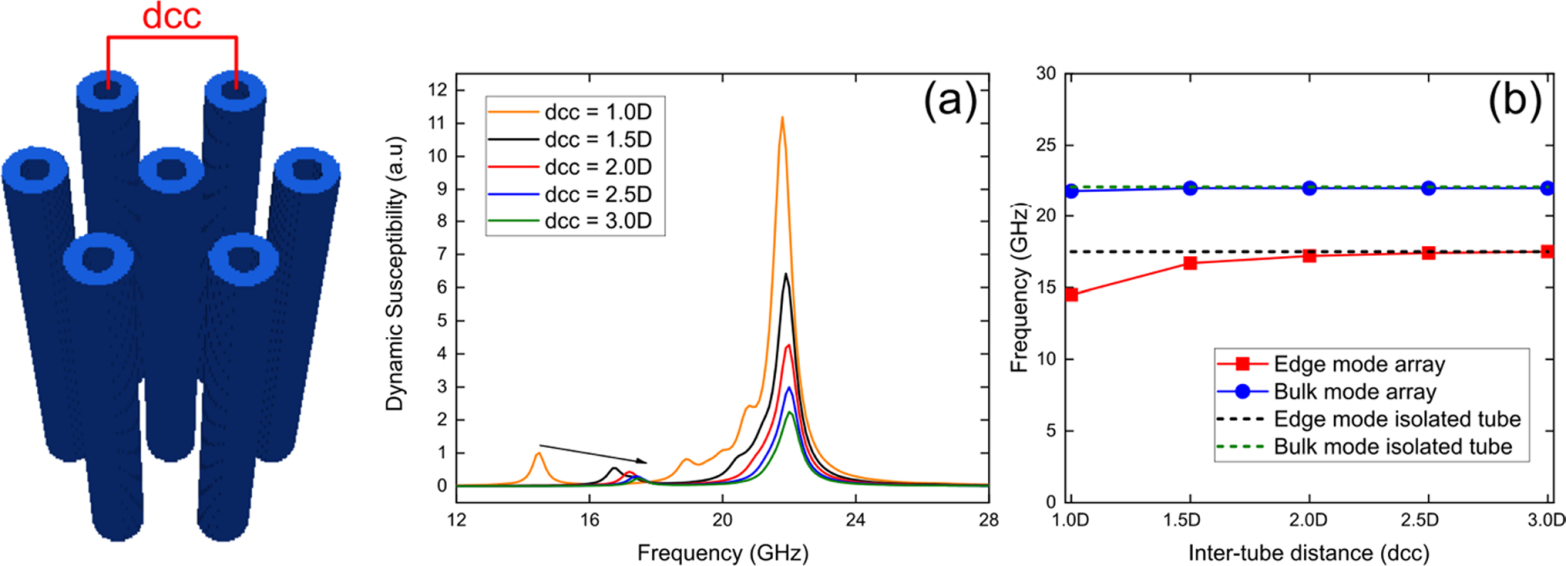
Dynamic susceptibility spectra for a hexagonal array consisting of seven 1000 nm long Fe_3_O_4_ nanotubes of *D* = 52 nm and *W* = 15 nm each one, for **a** five mean inter-tube distances taken between center to center (*d*_cc_) as a function of *D*, in the presence of an external magnetic field of *B* = 5.0 kOe along the *z*-axis. **b** Evolution of resonance modes, edge mode (red squares) and bulk mode (blue circles), as a function of the inter-tube distance

Finally, we investigated the dynamic susceptibility spectra for a hexagonal array consisting of seven 1000 nm long Fe_3_O_4_ nanotubes, each with a diameter of *D* = 52 nm and a wall thickness of *W* = 15 nm, as a function of the mean inter-tube distance taken between center to center (*d*_cc_). The simulations were performed in the presence of an external magnetic field of *B* = 5.0 kOe along the *z*-axis. From Fig. 4, we observe that the nanotube array displays the two resonance modes already seen for the single nanotubes. Figure 4b shows that the edge mode increases its resonance frequency with increasing inter-nanotube distance (*d*_*cc*_), from about 15 GHz for *d*_cc_ = 1.0*D* to ~ 17.5 GHz for *d*_*cc*_ = 2.5*D*, and remains constant for equal or larger values of *d*_cc._ Likewise, the bulk mode increases its respective resonance frequency with increasing *d*_cc_ and then becomes constant at a smaller dcc value than the edge mode case. Specifically, the bulk mode increases from to ~ 21.5 GHz for *d*_*cc*_ = 1.0D to ~ 22 GHz for *d*_cc_ = 1.5D, remaining constant for equal or larger *d*_cc_.

## Conclusions

In conclusion, through micromagnetic simulations we have been able to study the dynamic susceptibility of 1000 nm long Fe_3_O_4_ nanotubes by varying the diameter (*D*), the tube wall thickness (*W*), the magnitude of the external magnetic field (*B*) applied along the tube axis (*z*-axis) and the inter-nanotube distances in a Fe_3_O_4_ nanotube array.

For the entire range of geometric and magnetic parameters investigated, we found two well-defined modes, one at low frequency, edge mode, associated with the caps of the nanotubes, and another at high frequency, bulk mode, associated with the central area of the nanotubes. From the results obtained, we can conclude that the modes slightly decrease their resonance frequency as the diameter (*D*) of the nanotubes increases. In addition, we found that the edge mode significantly decreases its resonance frequency with increasing tube wall thickness (*W*), while the bulk mode resonance frequency practically does not change with this thickness. Besides, we obtained that the resonance frequency of both modes increases linearly with the magnitude of the external magnetic field (*B*) applied along the *z*-axis, which also produces a decrease in the intensity of the peaks. Finally, we showed that the edge mode for an array becomes constant from an inter-tube distance that is equal or larger than two and a half the nanotube diameter, while the bulk mode remains constant from an inter-tube distance equal or larger than one and a half the nanotube diameter.

These results suggest that these nanotubes could have potential applications in both electromagnetic interference shielding and microwave devices such as filters, isolators, and circulators, all of which require control of the resonant frequency in the GHz range.

## Supplementary Information

Additional file1 (DOCX 194 KB)

## Data Availability

The datasets generated during and/or analysed during the current study are available from the corresponding author on reasonable request.
