# Supplementary material for: Dynamic susceptibility of Fe3O4 nanotubes
Source: Discov Nano. 2023 Apr 7;18(1):61. doi: 10.1186/s11671-023-03841-5 (PMC10409952; doi:10.1186/s11671-023-03841-5)
Supplement: Supplementary file 1 — Additional file1 (DOCX 194 KB) [file 11671_2023_3841_MOESM1_ESM.docx]

**Dynamic susceptibility of Fe_3_O_4_ nanotubes**

**Enzo Fabrizio Pusiol^1^, Eduardo Saavedra^2^, Alejandro Pereira^3^, Juan Luis Palma^4,5^, Noelia Bajales Luna^1,6^, Juan Escrig^2,5*^**

^1^FAMAF, Universidad Nacional de Córdoba, 5000 Córdoba, Argentina.

^2^Department of Physics, University of Santiago de Chile (USACH), 9170124 Santiago, Chile.

^3^Department of Sciences, Faculty of Liberal Arts, Adolfo Ibañez University, 7941169 Santiago, Chile.

^4^School of Engineering, Central University of Chile, 8330601 Santiago, Chile.

^5^Center for the Development of Nanoscience and Nanotechnology (CEDENNA), 9170124 Santiago, Chile.

^6^CONICET, IFEG, Av. Medina Allende s/n, 5000 Córdoba, Argentina.

^*^ [juan.escrig@usach.cl](mailto:juan.escrig@usach.cl)


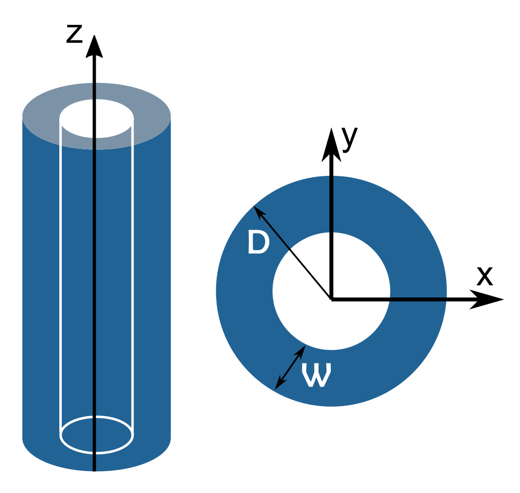


**Figure S1.** Schematic representation of an isolated magnetic nanotube. Representative geometric parameters are indicated.


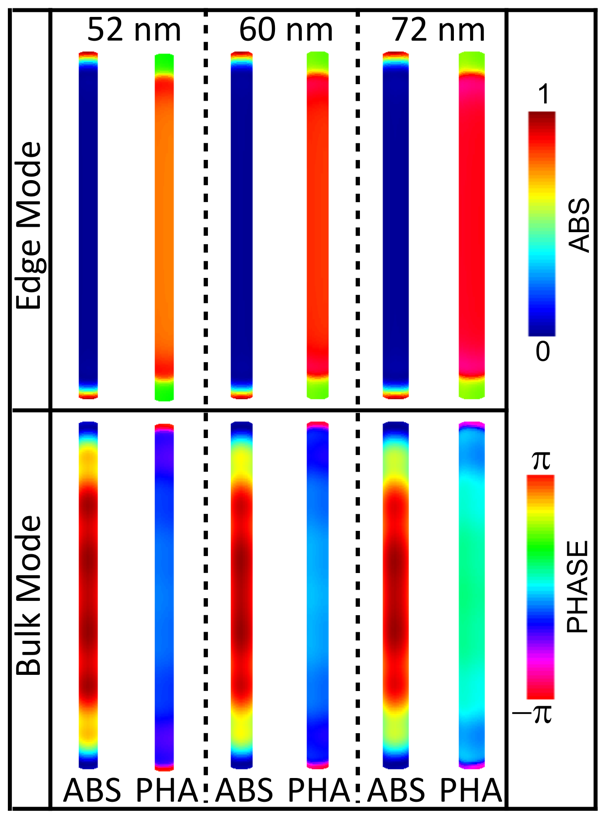


**Figure S2.** Spatial profiles of the resonant modes shown in **Fig. 1**.
